# High-Performance Flexible Ultraviolet Photodetectors with Ni/Cu-Codoped ZnO Nanorods Grown on PET Substrates

**DOI:** 10.3390/nano9081067

**Published:** 2019-07-25

**Authors:** Hafiz Muhammad Salman Ajmal, Fasihullah Khan, Noor Ul Huda, Sunjung Lee, Kiyun Nam, Hae Young Kim, Tae-Hyong Eom, Sam Dong Kim

**Affiliations:** Division of Electronics and Electrical Engineering, Dongguk University, Seoul 100-715, Korea

**Keywords:** Flexible UV detector, Ni/Cu codoping, ZnO nanorods, PET substrate, spectral responsivity

## Abstract

As a developing technology for flexible electronic device fabrication, ultra-violet (UV) photodetectors (PDs) based on a ZnO nanostructure are an effective approach for large-area integration of sensors on nonconventional substrates, such as plastic or paper. However, photoconductive ZnO nanorods grown on flexible substrates have slow responses or recovery as well as low spectral responsivity ***R*** because of the native defects and inferior crystallinity of hydrothermally grown ZnO nanorods at low temperatures. In this study, ZnO nanorod crystallites are doped with Cu or Ni/Cu when grown on polyethylene terephthalate (PET) substrates in an attempt to improve the performance of flexible PDs. The doping with Ni/Cu or Cu not only improves the crystalline quality but also significantly suppresses the density of deep-level emission defects in as-grown ZnO nanorods, as demonstrated by X-ray diffraction and photoluminescence. Furthermore, the X-ray photoelectron spectroscopy analysis shows that doping with the transition metals significantly increases the oxygen bonding with metal ions with enhanced O/Zn stoichiometry in as-grown nanorods. The fabricated flexible PD devices based on an interdigitated electrode structure demonstrates a very high ***R*** of ~123 A/W, a high on-off current ratio of ~130, and a significant improvement in transient response speed exhibiting rise and fall time of ~8 and ~3 s, respectively, by using the ZnO nanorods codoped by Ni/Cu.

## 1. Introduction:

Flexible sensors using inorganic or organic nanostructures have been employed for various sensing applications, such as wearable prosthetics [1], organs-on-chips [2], and noninvasive pathology [3]. For this reason, high-performance flexible ultraviolet (UV) photodetectors (PDs) using nanostructured semiconductor oxides are also emerging as a potential candidate for future commercial and military applications, such as pollution monitoring, secure space communications, water purification, and missile plume detection. Recently, much research has been done on flexible UV PDs using hydrothermally grown zinc oxide (ZnO) nanorods grown on plastic substrates [4,5]. However, in general, they reveal low crystallinity induced by the native defects emerging by a low thermal treatment during the post-annealing steps for hydrothermal growth of ZnO nanorods [6,7,8], thereby deteriorating the overall performance of PDs. Recently, control over the morphology, structure, and position of the nanorods has been demonstrated to improve the performance of PDs by tuning the experimental conditions of the growth process [9]; however, engineering the nanorod growth process via tuning the process parameters involves complicated device processing which can be neither reliable or reproducible in process condition. Therefore, the doping with transition metal impurities in ZnO nanorods has been studied as a viable alternative solution to improve the UV PD performance by orders of magnitude [10]. Furthermore, the ZnO nanostructure has emerged as a dominant host material for the doping of transition metals due to its versatile properties and potential applications. Apart from the UV sensor applications, metal-doped ZnO nanostructures have been investigated by many research groups recently for solar cells, light emitting diodes, water splitting, and hydrogen generation [11,12,13]. They demonstrated the impact of doping on the intrinsic defects, optical and electrical properties by varying the dopant concentrations in ZnO nanocrystalline structures.

Numerous bottom-up approaches have been explored for the preparation of doped and undoped ZnO nanorods, such as molecular beam epitaxy, thermal decomposition, sputtering, vapor phase epitaxy, spray pyrolysis, co-precipitation, and pulsed laser deposition [11,12,13,14]. Most of them either employ expensive and sophisticated laboratory facilities or are limited by high-temperature ZnO nanorods growth on organic substrates, such as textile fibers or plastic polymers. Therefore, a facile hydrothermal growth scheme for the synthesis of ZnO nanorods in aqueous solution has been extensively examined because of its reasonable thermal budget, reproducibility, and suitability for a scalable roll-to-roll fabrication [15]. Likewise, the synthesis of ZnO nanorods on organic substrates, for instance, polyethylene terephthalate (PET) as used in this work, needs to be accomplished at significantly low temperature because of their low thermal deformation temperature (~150 °C). Moreover, the hydrothermal technique provides a unique opportunity to dope as-grown nanorods with transition metals in a growth solution [16,17].

The *4s^2^* orbital of Zn in ZnO crystals contains two electrons, and they interact with and fill up the *2p*^6^ orbital of oxygen. For this reason, the stoichiometric ZnO with empty *4s*^2^ Zn orbital and completely filled *2p*^6^ O orbital ends up with an intrinsic semiconductor. However, ZnO grown hydrothermally is oxygen deficient in practice, which makes ZnO nanorods intrinsically *n*-type because of the ionization of excess *4s*^2^ electrons of Zn. It has been accepted that the unintentional *n*-type nature of as-grown ZnO is caused by the presence of Zn interstitials or oxygen vacancies; however, the matter of precise cause of *n*-type conductivity is still a debatable subject. For example, the cause for this can be related to the unintentional incorporation of shallow donor impurities, such as hydrogen which can be present in almost all growth conditions [18]. An undesirable UV sensing phenomenon is often observed from the PDs based on ZnO nanorods prepared in pristine condition because of the enormous *n*-type carrier concentration and quick recombination rate of photo-excited electron–hole pairs. Therefore, doping with transition metals, such as Mn, Ni and Cu, may resolve this critical problem and produce a desirable optoelectronic material quality for high-sensitivity UV sensor applications [19,20,21]. The presence of transition metal ions in a ZnO host lattice can modify the electronic and magnetic characteristics of ZnO because of the exchange interaction of the electrons in *d*-orbitals of transition metals with the electrons of ZnO in *s* and *p* orbitals. Recently, much effort has been focused on pristine ZnO nanostructures grown on plastic substrates, but little work has been reported on UV PDs based on transition metal doped ZnO nanocrystals [22,23]. In particular, the progress of research on PD devices fabricated using Ni/Cu codoped ZnO nanorods grown on plastic substrates has been very limited.

In this work, we synthesized two different UV PDs based on ZnO nanorods doped by Ni and Cu together or Cu-only using a hydrothermal method to compare the UV photoresponse with that of the devices based on undoped ZnO nanorods. Cu and Ni have been primarily chosen because: (i) ions of these dopants can swap the Zn ions without making any cationic vacancy, and (ii) they hold tetrahedral sites in the host lattice of ZnO in order to generate the required divergent physical properties. Among the various transition metals, Cu dopants are especially interesting because of their electronic shell structure is comparable to the chemical and physical properties of Zn. Cu impurities can improve the crystallinity of ZnO nanorods by suppressing the defects related to oxygen or zinc vacancies [24]. However, Ni dopants also have the advantage of the fair solubility of Ni^2+^ ions in ZnO tetrahedral coordinates because their ionic radius is comparable to that of Zn^2+^ [25,26]. The possible substitution of Zn^2+^ by Ni^2+^ can allow easy charge separation and transportation in ZnO nanorods.

In this study, we investigated the evolutionary change of nanorod crystalline quality pursued by addition of transition metal dopants into the ZnO nanocrystals. For this, we examined the morphological, structural, chemical, and optical characteristics of the nanorods grown atop the PET substrates. UV PDs with an interdigitated electrode (IDE) structure were fabricated in order to verify our analysis on the improvement of nanorod crystalline quality by the dopings, and important device parameters influenced by the nanorod material properties were measured and analyzed.

## 2. Experimental Procedure 

**Figure 1:**
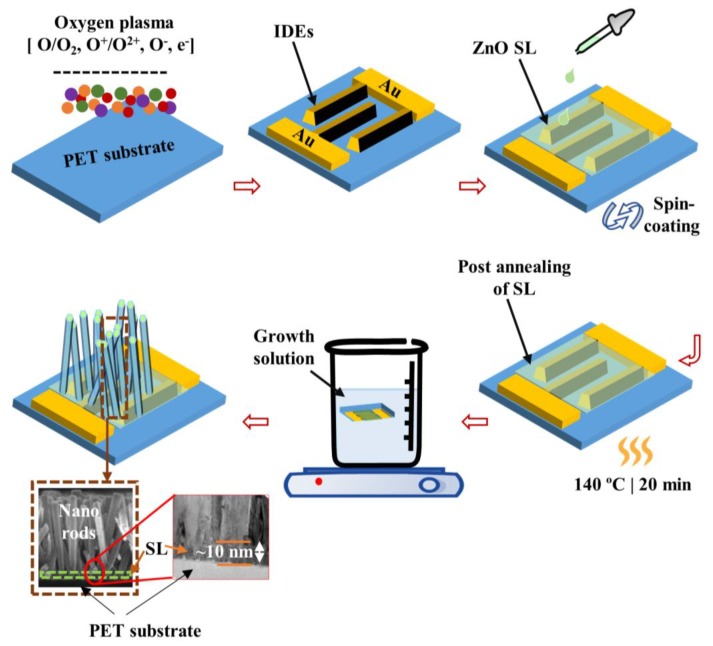
Schematic illustration of the process steps for zinc oxide (ZnO) nanorods hydrothermal growth. Cross-sectional scanning electron microscopy and transmission electron microscopy (TEM) views of as-grown nanorods are shown in the bottom-left insets.

To fabricate the IDE-type UV PDs based on ZnO nanorods, we used commercially available PET substrates of a 1.5 × 1.5 cm^2^ dimension. The device fabrication process flow was briefly illustrated in the schematics of Figure 1. All the chemical reagents (analytically graded with high purity >99%) used in this experiment were from Sigma-Aldrich, South Korea, and used without further purification. Before beginning the device fabrication, we first cleaned the PETs by sonication in ethanol and isopropyl alcohol and rinsed them with de-ionized (DI) water (8–10 min) sequentially to remove any organic contaminants from the surface. The substrates were dried in a clean room environment by purging with nitrogen gas followed by complete removal of moisture on a digital hot plate at 90–100 °C for 2 min. The device process starts with the fabrication of IDE patterns using Au/Ti metals and the subsequent spin coating of ZnO seed layer (SL), however, to attain a hydrophilic surface of the PET substrate, it is essential to achieve strong adhesion between the Au/Ti thin films and substrates. For this purpose, we pretreated the substrate surface by O_2_ plasma beforehand to improve the surface adhesion by converting the surface state of PETs to hydrophilic. In this treatment, plasma exposure was maintained in a reactive ion etcher system for 5 min at 100 sccm O_2_ flow rate, 100 W RF power, and 50 mTorr pressure. We then subjected the substrates to a photolithography system (Karl Suss, MA6 mask aligner, 365 nm) by employing a photoresist image-reversal technique to pattern IDEs and then loaded all patterned substrates into an electron-beam evaporator to deposit 100/30 nm Au/Ti films. After that, a metal lift-off was done in acetone using an ultra-sound bath sonicator to attain required IDE patterns on the PET substrates. 

**Figure 2:**
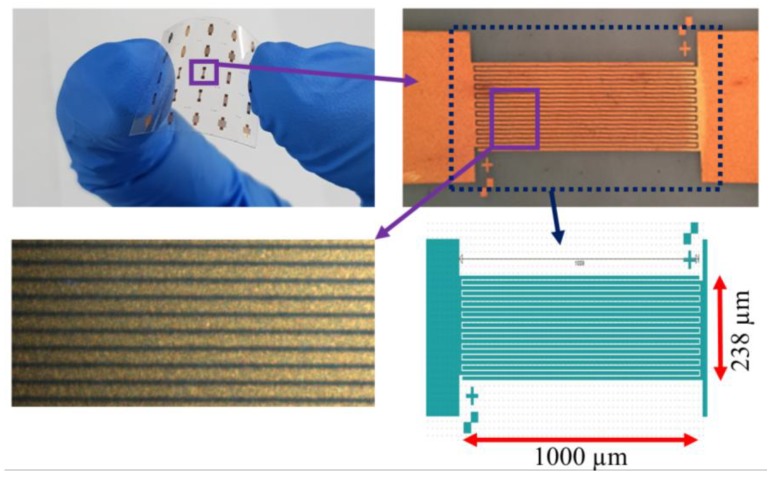
(**Top-left**) Fabricated flexible ultra-violet (UV) photodetectors(PDs) with interdigitated electrode (IDE) patterns. (**Top-right**) Magnified view of the fabricated PDs. (**Bottom-left**) Optical microscope image of ZnO nanorod-arrays grown on IDE structure. (**Bottom-right**) Layout of the IDE pattern for UV PDs.

Fabricated PDs on PET substrates with IDE patterns are shown in Figure 2. The IDE pattern has a dimension of 238 × 1000 μm^2^ with 20 fingers (10 μm width, 2 μm spacing) as depicted in the bottom-right layout of Figure 2. As shown in the micrograph (bottom-left of Figure 1) of nanorod-arrays grown atop the IDE structure, many ZnO nanorods are cross-linked between Au electrodes by forming nanorod-nanorod bridging junctions after the completion of process.

We adopted an aqueous solution route for the nanorods growth on the IDEs comprising a spin-coating step of ZnO SL and the subsequent nanorod growth step in a growth solution. To prepare the SL growth, we first produced a colloidal solution of 20 mm concentration of zinc acetate dehydrate [Zn(CH_3_COO)_2_·2H_2_O] in an organic solvent of *n*-propyl alcohol [C_3_H_8_O] and placed it in an ambient temperature for 8–10 h to achieve a homogeneous SL solution. The solution was spun at 3000 rpm for 30 s to deposit the SL on the substrate and was baked at 100 °C on a hotplate for 1 min. This spin-coating was successively repeated seven times to produce a final thickness of ~10 nm after post-bake as measured by our cross-sectional transmission electron microscopy (TEM) shown in a bottom-left inset of Figure 1. The SL-coated substrates were post-annealed on a hotplate at 140 °C for 20 min in an air ambient to evaporate any undesirable organic residuals and to form stable ZnO crystallites. Finally, we performed the growth of nanorods in the following way. For the growth of undoped ZnO nanorods, we prepared a homogeneous equimolar (25 mm molarity) solution of methenamine [C_6_H_12_N_4_] and zinc nitrate hexahydrate [Zn(NO_3_)_2_·6H_2_O] in a glass beaker containing 250 mL DI water and stirred the growth solution for 1 h to ensure complete mixing. The substrates were immersed in the growth solution upside down at a temperature of 80 °C for 4 h. In the cases of Cu doped and Ni/Cu codoped ZnO nanorods, we prepared two separate batches of solutions containing 25 mm equimolar C_6_H_12_N_4_ and Zn(NO_3_)_2_·6H_2_O. The growth solutions for Cu and Ni/Cu dopings were respectively provided by introducing 2 mm copper acetate monohydrate [(CH_3_COO)_2_Cu·H_2_O] and 2 mm of nickel acetate tetrahydrate Ni(OCOCH_3_)_2_.4H_2_O with 2 mm of (CH_3_COO)_2_Cu·H_2_O solutions in each batch. 

We examined the surface and cross-sectional morphologies of ZnO nanorods by field emission-scanning electron microscopy (FE-SEM, S-4800S Hitachi, Tokyo, Japan, 10 kV) and cross-sectional TEM (Hitachi, Tokyo, Japan, 9500 at 300 kV). The crystalline quality and preferred orientation of the nanorods were investigated by X-ray diffraction (XRD, D8 Advance spectrometer of Bruker, Billerica, MS, USA, AXS with Cu Kα 0.1540 nm radiation). Evolutionary change in stoichiometry and chemical bonding state of the nanorods depending on doping was observed by X-ray photoelectron spectroscopy (XPS, Jeol, Seoul, Korea) after cleaning the sample surface by ion milling (~10 nm) using a PHI 5000 Versa Probe (Ulvac-PHI) spectrometer and a monochromator Al Kα (1400 eV) anode (25.0 W, 15 kV). To evaluate the optical characteristics of the nanorod crystallites, we carried out photoluminescence (PL, Hitachi, Tokyo, Japan) spectroscopy (Bio MFP-3D with 325 nm He-Cd laser) and UV-Visible absorbance spectroscopy (model T-60, Labindia, India) at room temperature. Photo-response current-voltage (I-V) characteristics, real-time transient characteristics upon UV on-off, and spectral responsivity of the fabricated PDs were measured by our measurement setup. 

## 3. Results and Discussion

**Figure 3:**
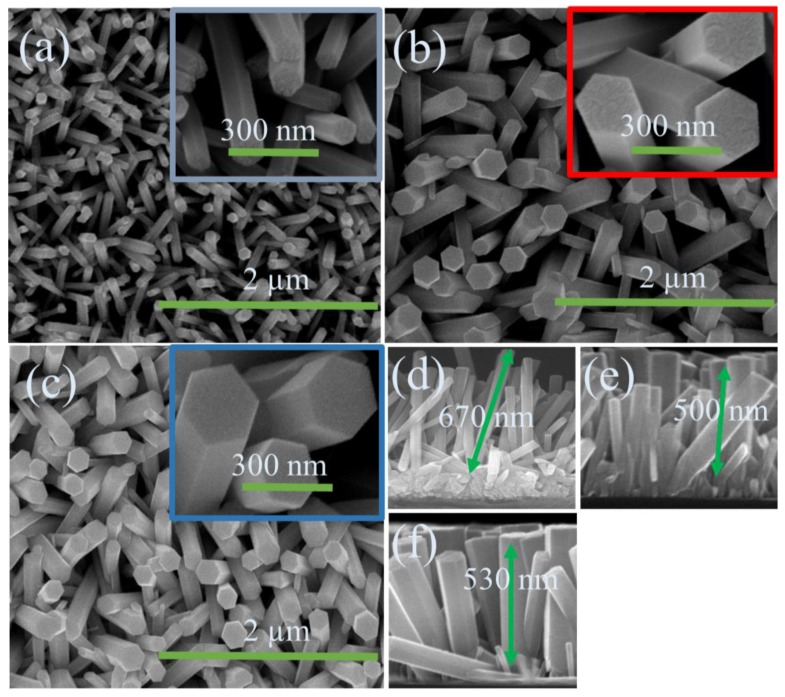
Scanning electron microscopy (SEM) top views of (**a**) undoped, (**b**) Cu doped, and (**c**) Ni/Cu codoped ZnO nanorods grown on polyethylene terephthalate (PET) substrates. Cross-sectional views of the ZnO nanorods grown under each condition are also shown in (**d**), (**e**) and (**f**).

The geometry and the surface morphology of undoped, Cu doped, and Ni/Cu codoped ZnO nanorods are shown in the SEM images of Figure 3a–c. As revealed in the top-view images, the nanorods were grown with fairly uniform distribution of diameters and the perfect hexagonal surface shape of wurtzite crystal structure. The measured average diameter was ~80 nm in the case of undoped ZnO nanorods, and it was also shown that the PET surface was uniformly covered with as-grown ZnO nanorod-arrays. On the other hand, the diameters of Cu doped and Ni/Cu codoped ZnO nanorod were increased up to ~150 and ~166 nm, respectively. Average length of the nanorod crystals was reduced from ~670 nm (undoped) to ~500 and ~530 nm in the case of Cu doping and Ni/Cu codoping, respectively. Similar phenomenon of lateral crystal coarsening was observed in a number of earlier experiments by incorporating either Ni or Cu into the ZnO nanorods grown by solution-based method on Si substrates [25,26,27,28,29], and this phenomenon is supposed to be caused by promoted lateral growth associated with higher bond energy of transition metal-O than that of Zn-O and reduced surface energy difference between polar and nonpolar planes [25,28]. 

**Figure 4:**
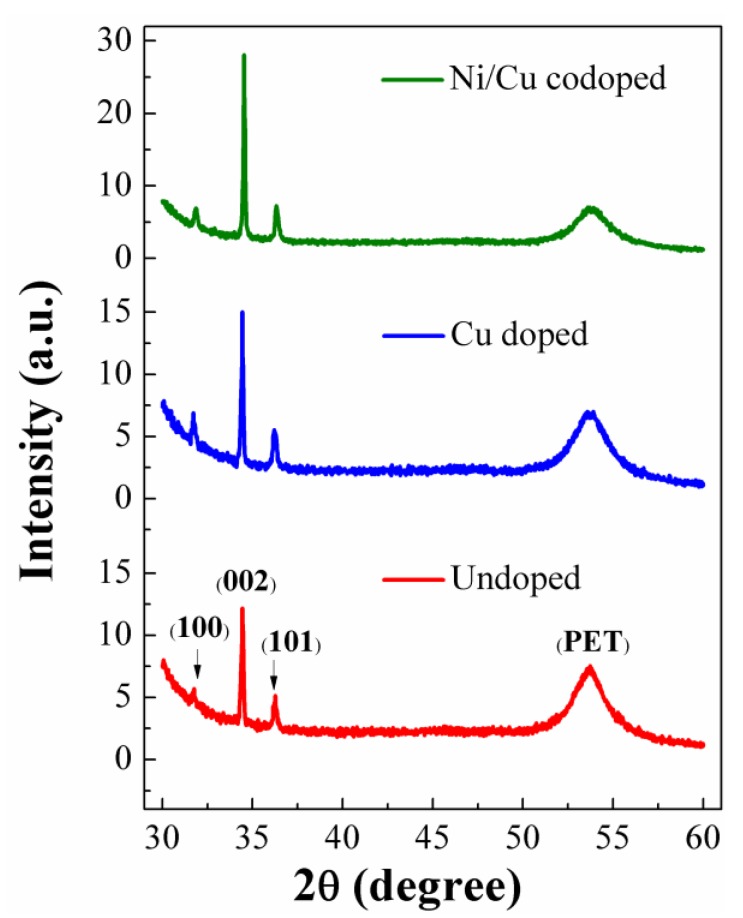
X-ray diffraction (XRD) 2θ patterns of as-grown ZnO nanorods.

The nanorods were also grown preferentially in the vertical direction with higher surface density, as shown in cross-sectional SEM images of Figure 3d–f, by the introduction of transition metal dopants into ZnO crystals. This SEM observation shows a good agreement with our XRD analysis. To examine the crystallographic structure and the degree of preferred orientation in growth direction, we performed a high-resolution XRD 2θ scan for the ZnO nanorods. As depicted in the XRD patterns of Figure 4, all the observed diffraction peaks were in good agreement with standard indexes of JCPDS data (36–1451 card number). The most dominant peak was (002) reflection at 2θ = 34.5° in all samples examined in this study. This result indicates that the intrinsic growth characteristics of hexagonal-wurtzite ZnO nanorod crystals is aligned to the *c*-axis with a growth direction normal to the substrate. The (002) peak intensity was improved after Cu doping or Ni/Cu codoping for the ZnO nanorods, which reveals the enhancement of (002) preferred growth orientation of our transition metal-doped ZnO nanorod crystals. Other reflections of relatively lower intensity from (100) and (101) planes were also observed at 31.7° and 36.2°, respectively. As investigated in many earlier reports [29,30], this (002) preferential growth of ZnO nanorod crystals is well known as an intrinsic property due to the lowest free surface energy (1.6 J/m^2^) of (002). In addition, no impurity traces or any other diffraction peak related to secondary phases from Cu, Cu_2_O, CuO, Ni, or NiO was detected within our XRD detection limit. This result suggests that Ni or Cu dopants used in this study did not form any new heterogeneous compound phases in the ZnO crystal but dissolved into the lattice matrix as a dopant [31]. 

We summarized in Table 1 the full width at half maximum (FWHM) at (002) diffraction peak with corresponding peak position (2θ), lattice constant *c*, and the calculated degree of orientation from XRD spectra for each sample. As shown in the table, the (002) FWHM was significantly decreased with Cu doping, and it was decreased to about 1/2 of the value of undoped sample after Ni/Cu codoping, in particular. This proposes that our transition metal doping, especially Ni/Cu codoping, plays a significant role in improving the crystalline quality of our ZnO nanorods crystals grown on PET substrates. Furthermore, the (002) 2θ positions of Cu doped and Ni/Cu codoped samples showed slight shifts (~34.46° and ~34.48°) toward higher angles compared to undoped sample (~34.45°). This observation agrees with the previous experimental reports of possible replacements of Zn^2+^ by either Cu^2+^ or Ni^2+^ in the host ZnO lattice without changing the crystal structure. The minor shift in the position of (002) reflection arises because the radii of Ni (0.69 Å) and Cu (0.73 Å) are smaller than that of Zn^2+^ (0.74 Å) ions [25,32]. This phenomenal substitutions of Cu or Ni dopants in the ZnO wurtzite hexagonal lattice can also induce a change in lattice parameter *c* because of compact CuZnO_4_ units or shorter bonds of CuZn–O in Cu doped Zn-Ni-O material [33]. The value of *c* for the hexagonal structure can be calculated using the following expression [34]:

(1) $$\frac{1}{d^{2}}=\frac{4}{3}\left(\frac{h^{2}+hk+k^{2}}{a^{2}}\right)+\frac{l^{2}}{c^{2}}$$

where *d* is the spacing between the lattice, *h*, *k*, and *l* represent the miller indices, and *a* and *c* are the cell lattice parameters. The 0.19% decrease in lattice constant ***c*** with the introduction of Cu or Ni/Cu dopants may arise from the replacement of Zn^2+^ ions by either Cu^2+^ or Ni^2+^ and their corresponding ionic-radii mismatch [35]. Furthermore, The degree of preferred orientation *F(hkl)* was calculated using the following equation [36],

(2) $$F\left(hkl\right)=\left(P\left(hkl\right)-P_{0}\left(hkl\right)\right)/(1-P_{0}\left(hkl\right)$$

where P(hkl)=I(hkl)/ΣI(hkl), $P_{0}\left(hkl\right)=I_{0}\left(hkl\right)/\Sigma I_{0}\left(hkl\right)$, I(hkl) is the measured intensity of the diffraction peak reflected from the (*hkl*) plane, and $I_{0}\left(hkl\right)\;$is the intensity of the reference peak of the (*hkl*) plane given by JCPDS (36–1451). The *F (002*) calculations in Table 1 show that the *c*-axis alignment of synthesized nanorods was improved after Cu or Cu/Ni doping. 

**Figure 5:**
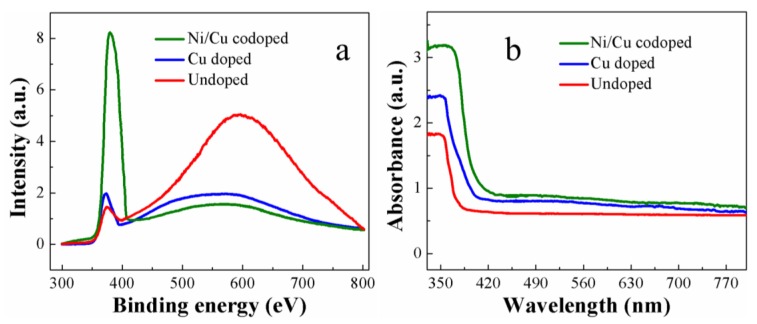
(**a**) Photoluminescence (PL) spectra and (**b**) ultraviolet (UV)/visible spectra of as-grown ZnO nanorods measured at room temperature.

Structural defects such as O vacancies or metal interstitials can significantly influence the optical characteristics of oxide nanostructures. As-grown wurtzite nanostructure crystallites of ZnO exhibit, in general, pretty good optoelectronic properties among the wide band-gap semiconductors; nonetheless, the luminescence properties of Ni/Cu codoped ZnO nanocrystals grown on plastic substrates prepared at a low temperature have been rarely explored. PL characterization at room temperature is one of the most efficient ways to evaluate the defect states and crystalline quality of synthesized ZnO nanorod crystals. Figure 5a shows the measured PL spectra, and they have two prominent emission zones of a sharp UV emission region with a span of 366–400 nm and the other region of broad-band emission in the visible range (400–800 nm). The UV emission spectra of near-band emission (NBE) originates from the band-to-band excitonic recombination in ZnO crystals [37]. Significantly improved UV emissions recorded from the transition metal-doped nanocrystals can represent their enhanced crystalline quality; especially in the case of Ni/Cu codoping, a very pronounced increase in NBE was shown in the spectra. Peak positions of NBE in each case of undoped, Cu doped, and Ni/Cu doped ZnO nanorods were 372, 371, and 376 nm, respectively. This slight difference in the emission wavelength is known to arise from various phenomena, such as the localization of charge carriers, electron phonon coupling, and lattice distortion in ZnO crystals [33,38].

The broadband spectra of visible emission in a range of 400–800 nm are to be attributed to a deep-level emission (DLE) caused by the recombination of photo-excited charges of many different types of intrinsic defects, such as oxygen vacancies and Zn interstitials, present in the nanocrystals. The precise nature of these defects corresponding with each DLE location is still a debatable subject [39,40,41]; however, the DLEs from the materials grown by hydrothermal method, especially at low process temperature, exist in general. This is because, during solution-based processes such as hydrothermal growth, a large amount of residual organic materials from the growth solution can penetrate into the crystal and produce deep-level defects which cannot be easily eradicated at low process temperature. External transition metal doping for the ZnO nanorod crystals is one efficient way to control the defect emissions as reported in many earlier studies [42,43,44,45,46]. In this study, the visible emissions were also greatly suppressed by ~1.6 times after Ni/Cu codoping compared to the emission from undoped sample as shown in Figure 5a. The intensity ratio between the NBE peak (*I_UV_*) and DLE peak (*I_VIS_*) can be employed to estimate the degree of intrinsic defect formation in the synthesized ZnO nanorod crystals. *I_UV_*/*I_VIS_* ratios of our Ni/Cu codoped and Cu doped nanorods were ~5.2 and ~1.1, respectively, which are much higher than that (~0.3) of undoped ZnO sample.

Figure 5b illustrates the optical absorption spectra of the ZnO nanorod crystals measured at room temperature using a UV-Visible spectrometer. As shown in the spectra, the absorption of Cu doped or Ni/Cu codoped ZnO below cut-off wavelength was much higher than that measured from undoped ZnO nanorod crystals. The higher UV absorption in transition metal-doped ZnO nanorods could arise from a variety of reasons, such as the strain caused by doping and size effect of the nanorods [42]. In our case, the increases in the diameter of nanorod crystal and in the surface coverage of ZnO material on the surface by transition metal doping, as shown in the Figure 3, are supposed to be the main causes of the increase in absorption [23]. Another reason for the increase of absorption can be related with the existence of larger size of ZnO nanorods by Cu/Ni codoping, resulting in much photon absorption in the way of Mie light scattering which is maximized when the scattering particles have a diameter similar to or larger than the wavelength of the incident light [43]. Furthermore, the shift in the band-edge of doped ZnO nanorods indicates the incorporation of Cu^2+^ and Ni^2+^ into ZnO host lattice. The considerable red shift in the absorption band-edge of Ni/Cu codoped ZnO nanorods was observed due to the differences in *sp-d* exchange interactions between the localized *d*-electrons and the band electrons of Ni^+2^ [33]. 

**Figure 6:**
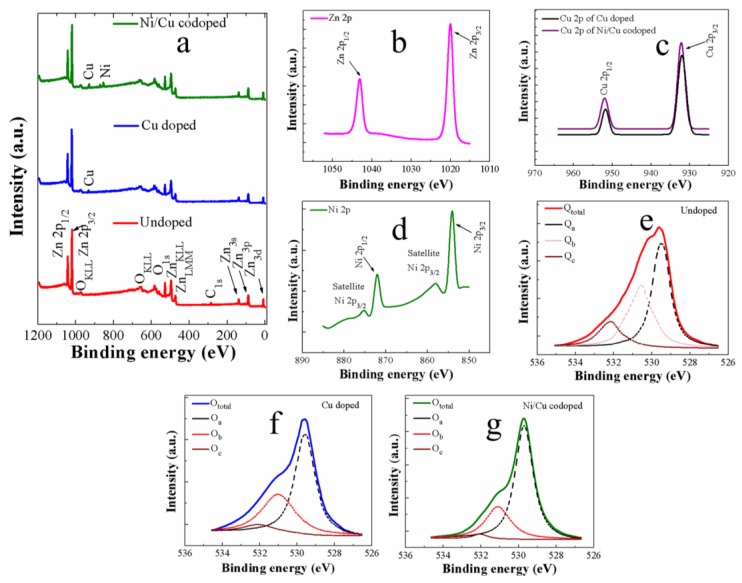
(**a**) Wide-scan X-ray photoelectron spectroscopy (XPS) spectra of the ZnO nanorods. High-resolution spectra of (**b**) Zn-2p (undoped), (**c**) Cu-2p (Cu doped and Ni/Cu codoped), and (**d**) Ni-2p (Ni/Cu codoped) core-levels measured from the ZnO nanorods. High-resolution O-1s core level spectra obtained from the ZnO nanorods prepared with (**e**) no doping, (**f**) Cu doping, and (**g**) Ni/Cu codoping are deconvoluted into three distant satellite peaks.

Wide-scan spectra of XPS carried out in a binding energy (BE) of 0–1200 eV for all synthesized nanorod crystals are shown in Figure 6a. All vital constituents of Zn, O, and externally added impurities of Cu and Ni showed their corresponding photoemission peaks of various core-levels and spin-orbital splittings along with Auger peaks, and limited traces of carbon were also observed at 285 eV. Two explicit core-level Zn peaks in the XPS spectra were indicated as Zn-2p_3/2_ (1020.8 eV) and Zn-2p_1/2_ (1043.9 eV) separated by spin-orbital splitting [see Figure 6b]. Symmetric shapes of these photoemission peaks, peak locations, and spin orbital splitting value of 23.1 eV of the Zn-2p doublet support the existence of the Zn^2+^ chemical state in the stoichiometry of ZnO in all cases of the samples. The Zn spectra measured from three different samples were almost similar to each other; therefore, the spectrum of undoped sample was shown in Figure 6b as a representative example. However, the Zn-2p_1/2_ peak in Ni/Cu codoped sample was shifted slightly toward higher BE, which is associated with a higher state of oxidation of the Zn in ZnO matrix. 

Figure 6c,d illustrate clear core levels of Cu-2p and Ni-2p, respectively, from the transition metal-doped samples. In the cases of Cu doped or Ni/Cu codoped samples, a split of Cu-2p photoemission into Cu-2p_3/2_ (933.09 eV) and Cu-2p_1/2_ (953.1 eV) was clearly observed in the spectra. Cu is cationic in the Cu doped samples as reported in the literatures [44,45,46]. The oxidation state of Cu dopants in ZnO nanorod crystals results from most probably either Cu^2+^, Cu^+^, or both, and no other distinctive photoemission related to the secondary phase such as CuO_2_ was detected from the Cu doped samples. A slight shift (~0.2 eV) toward higher energy in core level spectra of Cu-2p of Ni/Cu codoped nanorods (see Figure 6c) was shown, and this demonstrates that the occupation probability of Cu^2+^ valance state by Cu dopants is more dominant in Ni/Cu codoped sample as the higher binding energy of Cu-2p spectra is closer to Cu^2+^ state. A core-level splitting of Ni-2p_3/2_ and Ni-2p_1/2_ was also detected at 854.1 and 872.2 eV, respectively, with two corresponding satellite peaks at 857.5 and 875.7 eV. Two prominent Ni peaks observed in the Ni/Cu codoped samples were adjacent to that of the Ni^2+^ ions [47], and they confirm the presence of Ni^2+^ ions in the host lattice of ZnO. The satellite peaks are also observed due to the trivalent Ni^3+^ cations.

By investigating the asymmetric O-1s peaks in depth, it is possible to grasp the detailed information for the contribution of oxygen bindings in the ZnO crystals. We deconvoluted the O-1s peak into three satellite individual peaks of O_a_, O_b_, and O_c_ as shown in Figure 6e–g. Among them, a peak at the lowest BE (~529.7 ± 0.2 eV), symbolized as O_a_, is known to originate from the O^2−^ ions establishing bonds with the metal ions (Zn^2+^, Cu^2+^, Ni^2+^) in the wurtzite structure of ZnO. Since O_a_ is an excellent measure of stoichiometric oxygen existence in the ZnO crystals, we estimated the stoichiometry of each synthesized nanorod sample by estimating ʃO_a_**/**ʃZn, where ʃO_a_ and ʃZn respectively represent the peak curve integration of O_a_ and Zn in the spectra. It was clearly shown in Table 2 that the percentage contribution of O_a_ in O_total_ (O_total=_ O_a_ + O_b_ + O_c_) shows the maximum in the case of Ni/Cu codoped ZnO nanorods. Each percentage value of O_a_, O_b_, and O_c_ listed in Table 2 was estimated by ʃO_x_/ ʃO_total_, where ʃO_x_ can be obtained by curve integration of the O-1s peak (*x* = a, b, c), and ʃO_total_ is the summation of curve integrations for each O-1*s* satellite peak. Even though our ZnO crystals are oxygen deficient in nature, the atomic ratio of O_a_/Zn was significantly improved by transition metal doping, and the highest value of 0.78 was shown from the Ni/Cu codoped sample. This increase in O_a_ contribution can be attributed to the stronger bonding characteristics of metal-oxygen in Cu doped and Ni/Cu codoped nanorods because the electronegativity (χ) of Ni (χ  =  1.91) and Cu (χ  =  1.90) are higher than that of Zn (χ  =  1.65) [48,49]. The medium BE component of O_b_ (~531 ± 0.2 eV) is known to emerge from O^2−^ ions in the oxygen-deficient regions (where oxygen vacancies are present) of the ZnO matrix. The O_c_ component of higher BE (~532 ± 0.09 eV) is principally associated with chemisorbed oxygen or -OH species on the surface of ZnO nanocrystals during the growth of nanorods in organic solutions [50,51]. As summarized in Table 2, the presence of O_c_-related defects was at a minimum in transition metal-doped nanorods. Another reason why O_c_ contribution is higher in the case of undoped ZnO sample can be a higher surface-to-volume with longer average length and smaller average diameter of the nanorod crystals [52].

Based on the findings of our material characterizations, we fabricated UV-PDs of IDE patterns on the PET substrates. Current-voltage (I-V) characteristics, transient time response, and spectral responsivity were measured from the PDs. We carried out the photo-response characterizations of our flexible PDs using a 300 W wide-band (200–800 nm) Xenon lamp (model 300XF-R1) as an input light source. The source light was filtered into a single wavelength of 350 nm using a monochromator (CM11 1/4 m) with a grating of 2400 lines-per-mm. The output light from the monochromatic source was directed onto the surface of the samples, and the PD samples were probed using a Keithley source measurement unit with two-input probe station. The on/off of the input UV light for transient measurement was controlled by a programmable electronic shutter (model 71,445). The whole measurement setup was housed in a black box to prevent any influence of ambient light. 

**Figure 7:**
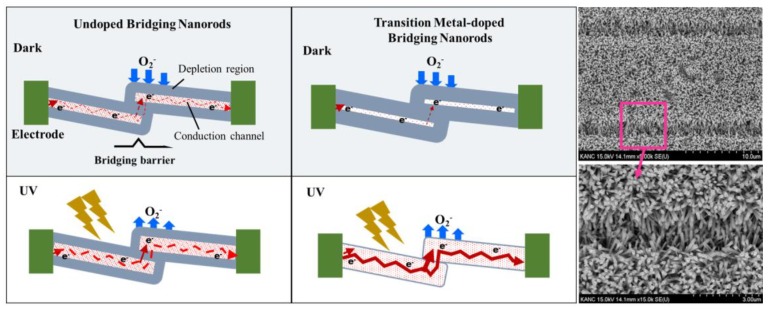
**Left:** Schematic illustrations of the carrier generation and transport processes in the bridging ZnO nanorods (undoped and transition metal-doped). **Right:** SEM images of bridging ZnO nanorods grown on the PET substrates fabricated with Au IDE pattern.

The UV sensing mechanism of photoconductive PDs based on ZnO nanorods is associated with the chemisorption of atmospheric oxygen at the surface of ZnO nanorods grown on IDE structure [53]. As-grown ZnO nanorods are *n*-type in nature because of various intrinsic defects, such as oxygen vacancies [10,54]. In the dark condition, oxygen molecules from the atmosphere tend to trap the free electrons present in the conduction band of *n*-type ZnO nanorods [55] and get adsorbed at the surface of the nanorods by $[O_{2}+e^{-}\;\overset{\;}{\to }\;O_{2}^{-}{}_{adsorbed}$], as illustrated in Figure 7. Consequently, the electron concentration at the surface of nanorod is reduced with the expansion of low conductivity surface depletion layer. 

**Figure 8:**
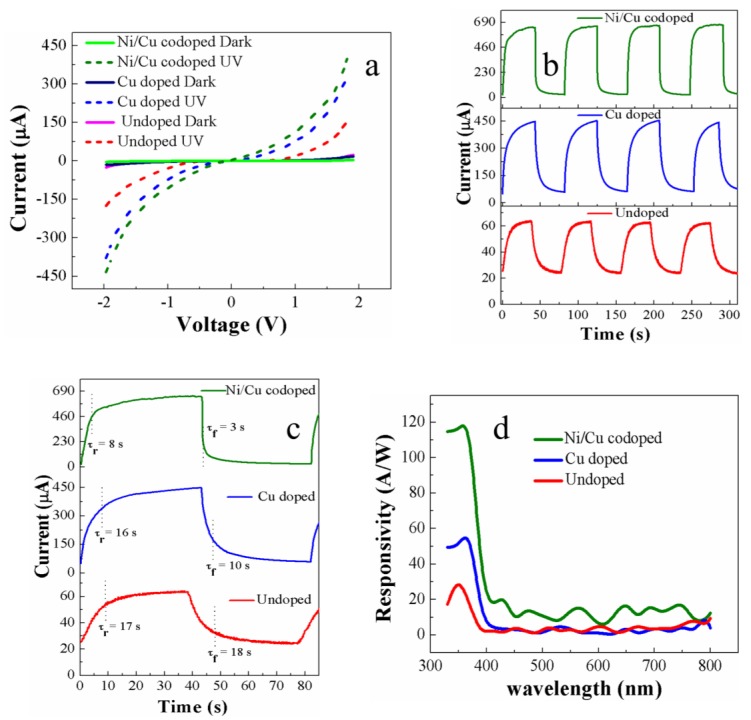
(**a**) Current-voltage (I-V) characteristics, (**b**) photo-response transient characteristics, (**c**) magnified views of single-cycle transient, and (**d**) spectral responsivities of PDs with undoped, Cu doped, and Ni/Cu codoped ZnO nanorods.

When Cu and/or Ni dopants are introduced, they tend to drop the free electron concentration, *n,* in the conduction band because of the reduced oxygen vacancies in the ZnO nanorod crystal as shown in our results of PL and XPS analysis and also in the report of *R*. Shabannia [24]. The surface depletion layer thickness, δ of the nanorods is given by [9]:

(3) $$\delta =L_{D}{\left(\frac{eV_{s}}{kT}\right)}^{1/2}$$

(4) $$L_{D}={\left(\frac{\varepsilon_{o}kT}{e^{2}n}\right)}^{1/2}$$

where *L_D_* is the Debye length, *V*_S_ is the adsorbate-induced band bending, *e* is the electron charge, *kT* is the thermal energy, and *ε_o_* is the permittivity. The surface depletion layer of the transition metal-doped ZnO nanorod will then grow thicker than that of undoped one. The conduction channels in our IDE PDs include nanorod-nanorod junctions. Electrons must overcome the bridging barrier to transport from one nanorod to another as shown in the left of Figure 7. These barriers are formed by the surface depletion layers. The channel conductance, *G*, of the bridging ZnO nanorods is influenced by not only *n* but also dimension of the conduction channel formed inside the nanorods, and this relationship [9] can be expressed by:

(5) G=neµπ(D−2δ)/4

where μ is the electron mobility, *D* represents the diameter of the nanorod, and l is the length between the two electrodes. However, more than this dimensional effect of conduction channel, the surface depletion regions in each nanorod act as barriers preventing the electron transfer through the nanorod-nanorod bridge since the current has to flow through this bridging barriers of connected nanorods. Therefore, when the diameters of the nanorods exceed the Debye length, such as in our case, the dominant bottleneck of charge transport in our PDs will be the nanorod-nanorod junction. Figure 8a shows the lowest dark current, *I_dark_*, of ~3.1 µA (at 2 V) in the case of the PDs with Ni/Cu codoped nanorods. On the other hand, much higher *I_dark_* of ~17.2 and ~25 µA were measured at 2 V from the PDs with Cu doped and undoped nanorods, respectively. The lowest *I_dark_* measured from the PDs with Ni/Cu codoped nanorods of the largest nanorods diameter supports our premise that the charge transfer in our PDs is controlled by the surface depletion layer at the nanorod-nanorod bridge.

Electron-hole pairs are generated by optical absorption under UV light illumination, and the generated holes recombine with the electrons trapped by the oxygen adsorbed [18]. This leads to a desorption process of the O_2_ molecules by [$O_{2}^{-}{}_{adsorbed}+h\;\overset{\;hv\;}{\to }O_{2}$] (see Figure 7). At the same time, the surface depletion region will keep shrinking if holes can be continuously supplied to the surface. Photocurrent, *I_UV_* (at 2 V), measured from the PDs with Ni/Cu codoped, Cu doped, and undoped nanorods were ~402, ~320, and ~153 µA, respectively, are shown in Figure 8a. Trapping of carriers under dark and detrapping them under illumination by the defect states can be responsible for higher *I_UV_* in PDs with hydrothermally grown transition metal-doped ZnO nanorods than that with undoped nanorods as claimed by S. Sarkar et al. [56]. According to their interpretation, the photo-excited carriers can rapidly recombine through many deep-level defects present in hydrothermally grown ZnO nanorods. However, it was also found that the introduction of transition metal dopants can significantly reduce the deep-level defects, thereby eventually minimizing the loss of photo-excited carriers with a remarkable increase in photocurrent. It was also reported by West et al. [57] that the Cu doping in ZnO forms neutral complexes that can be ionized by a photon of energy greater than 1.45 eV. This result also supports that the photo-excited electron concentration in the conduction band of the transition metal-doped samples is higher than that in undoped samples.

Another possible cause for enhanced photocurrent in transition metal-doped PDs is the minimized thickness of bridging barrier as illustrated in Figure 7. When Cu and/or Ni are doped into ZnO nanorods, as many photo-excited holes as possible are freed from the recombination or trapping by deep-level defects, thus continuously shrinking the surface depletion layer on the nanorod surface. Assuming that the tunnel current is an exponential function of the bridging barrier thickness, the current will be very sensitive to small change in the barrier. A great increase of the UV-to-dark current ratio *(I_UV_*/*I_dark_)* up to ~130 was obtained from the PDs with Ni/Cu codoped nanorods at 2 V, while a much lower *I_UV_*/*I_dark_* of ~6 was measured from the PDs with undoped nanorods.

One vital performance parameter for the PDs is the real-time transient response to estimate the rise-up (response) time and fall-down (recovery) time under UV illumination (at 350 nm) on and off, which are defined as time intervals for the *I_UV_* to rise up to 90% of maximum saturation value under UV turn-on and for the *I_UV_* to fall off by 90% from maximum value under UV turn-off, respectively. Overall transient speed of our UV PDs depends upon the process of adsorption or desorption kinetics of O_2_ on the ZnO nanorod surface; therefore, this surface kinetics is governed by the density of the defects near the surface as well as the partial pressure of atmospheric oxygen [56]. First, the improvement in response time of our PDs is deeply associated with the desorption kinetics of O_2_. The incorporation of Cu and/or Ni in ZnO nanorods increases the photo-generated holes, which move toward the surface and facilitate the neutralization of the ionized O_2_ molecules, due to significant reduction in the concentration of traps near the surface, thereby leading to a faster desorption from the ZnO surface. The response times measured from the PDs with Ni/Cu codoped and Cu doped nanorods were ~8 and ~16 s, respectively, which are far less than those (~17 s) from the PDs with undoped nanorods, as shown in Figure 8b,c. Due to the reduction in deep level defects and the shrinking of depletion layer in Cu or Ni/Cu doped nanorods, the transport rate of photo-generated holes toward the surface of ZnO nanorods can improve with the rise time which is associated with effective desorption of O_2_. In the case of PDs with undoped nanorods, the photo-generated charge carriers fill the traps first and photocurrent reaches a maximum (saturation) after all of the traps are filled which causes a delay in reaching the maximum photocurrent [58]. On the other hand, the improvement in recovery process of Cu or Ni/Cu doped nanorods is directly associated with O_2_ adsorption. The deep-level defects on the surface of transition metal-doped nanorods are considerably reduced, and this results in lowering the surface band bending, thereby increasing the diffusional flux of electrons toward the surface of nanorods to produce adsorbed O_2_^-^ ions with the reduction of electron energy barrier [50]. 

**Figure 9:**
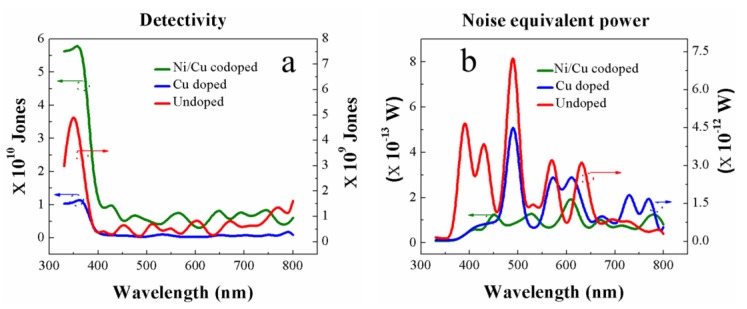
(**a**) Specific detectivity and (**b**) noise equivalent power as functions of radiant light wavelength.

Another important figure of merit to evaluate the PD performance is spectral responsivity ***R*** defined as follows [59].

(6) $$R=\frac{I_{UV}-I_{dark}}{P_{in}}$$

where *P_in_* represents the power of incident light on the active area of the fabricated device. Figure 9d shows the measured spectral responses of IDE-based UV-PDs with an effective device area of 0.238 mm^2^ at a bias voltage of 2 V under a light intensity of 140 µW/cm^2^. The PDs with Ni/Cu codoped nanorods showed a very high ***R*** of ~123 A/W at 370 nm. This high ***R*** value is mainly due to an excellent quantum efficiency of the PDs, and it arises from the fact that most of the electron-hole pairs generated during UV irradiation are collected into photocurrent with the minimized recombination taking place by defect levels in the high crystalline-quality doped nanorods. Another reason for the increase of ***R*** is the enhanced absorption related with the existence of larger size of ZnO nanorods by Cu/Ni codoping by Mie light scattering as discussed earlier. In conclusion, the ***R*** value measured from the PDs with Ni/Cu codoped nanorods is the best performance among the recently investigated UV sensors [60,61] based on ZnO nanostructures hydrothermally grown on flexible substrates.

The performance of our PDs was also evaluated by specific detectivity, ***D*** *, and noise equivalent power, NEP, normalized per square root of the device area which is equal to the reciprocal of detectivity. NEP is defined as the signal power that gives a signal-to-noise ratio of one in a one hertz output bandwidth. Therefore, this value is a measure of the weakest optical signal that can be detected, and it is desirable to have a NEP as low as possible. ***D*** * is defined as follows [62]

(7) $$D^{\;*}=\sqrt{A}R\;/\sqrt{2qI_{dark}}$$

where *A* represents the active device area and *q* is the charge of an electron. ***D*** * versus wavelength estimated at a bias voltage of 2 V and a light intensity of 140 mW/cm^2^ of our IDE-based UV-PDs are shown in Figure 9a. The highest D* of ~6 × 10^10^ Jones (cm Hz^1/2^/W) was obtained at 370 nm from the devices with Ni/Cu codoped ZnO nanorods. Despite that our PDs were fabricated at a very low temperature −150 °C to accommodate the thermally unstable plastic substrates, the measured detectivity was quite high and even comparable with those measured from the devices of Ni doped ZnO nanoparticles on glass (~3.7 × 10^10^ Jones) [28] and Mn-doped ZnO films on quartz (~1.6 × 10^10^ Jones) [63] prepared at much higher process temperatures. Figure 9b shows the NEPs of the fabricated PDs, and a very low noise power of ~8 × 10^−15^ W was measured at 370 nm from the devices with Ni/Cu codoped ZnO nanorods. 

## 4. Conclusions

We performed a comparative study on the performance of the IDE structure-based UV PDs fabricated on PET substrates with ZnO nanorods doped by Cu or Ni/Cu with the devices prepared with undoped nanorods. In conclusion, the UV-PDs based on Ni/Cu codoped nanorods exhibited significantly improved device performance in terms of spectral responsivity of (~123 A/W), on-off current ratio of (~130), and current rise and fall times (~8 and ~3 s) in transient response compared to those of devices fabricated by using undoped or Cu-only doped nanorods. As shown in our surface characterizations of SEM, XPS, PL and XRD, this improvement is due to the evolutionary change in morphology and crystalline quality of the nanorod crystals grown through Ni/Cu codoping with the enhancement in O/Zn stoichiometry, (002) preferred orientation, and the reduction of visible emission defects. This doping technique using a low-temperature ZnO nanorod synthesis method is expected to be used to improve the performance of PDs manufactured on flexible plastic substrates which can be an inevitable solution in the future because of their unique benefits such as high resistance to impact damage, transparency, transportability, and low cost.

## Figures and Tables

**Table 1:** XRD key parameters extracted from undoped, Cu doped, and Ni/Cu codoped ZnO nanorod crystals.

Sample	(002) 2θ Position (°)	FWHM (°)	*c* (Å)	Degree of (002) Orientation
Undoped	34.45	0.19	5.20	0.45
Cu doped	34.46	0.13	5.20	0.55
Ni/Cu codoped	34.48	0.09	5.19	0.66

**Table 2:** Atomic percentage values of Cu and Ni, percentage values of individual O-1s satellite peaks, and O/Zn atomic ratios of the nanorod crystals estimated by XPS deconvolution analysis.

Sample	Cu at %	Ni at %	Oa (%)	Ob (%)	Oc (%)	O/Zn Ratio
Undoped	0	0	47.74	38.31	13.11	0.63
Cu doped	0.58	0	56.81	32.79	9.98	0.71
Ni/Cu codoped	0.47	0.26	62.13	30.73	8.87	0.78
